# The Chk1 inhibitor SAR-020106 sensitizes human glioblastoma cells to irradiation, to temozolomide, and to decitabine treatment

**DOI:** 10.1186/s13046-019-1434-2

**Published:** 2019-10-21

**Authors:** Ina Patties, Sonja Kallendrusch, Lisa Böhme, Eva Kendzia, Henry Oppermann, Frank Gaunitz, Rolf-Dieter Kortmann, Annegret Glasow

**Affiliations:** 1 Department of Radiooncology, University of Leipzig, Stephanstraße 9a, 04103 Leipzig, Germany; 2 Institute of Anatomy, University of Leipzig, Liebigstraße 13, 04103 Leipzig, Germany; 3 Department of Neurosurgery, University of Leipzig, Liebigstraße 20, 04103 Leipzig, Germany

**Keywords:** Glioblastoma, Chk1 inhibitor, SAR-020106, Decitabine, Irradiation, Temozolomide

## Abstract

**Background:**

Glioblastoma is the most common and aggressive brain tumour in adults with a median overall survival of only 14 months after standard therapy with radiation therapy (IR) and temozolomide (TMZ). In a novel multimodal treatment approach we combined the checkpoint kinase 1 (Chk1) inhibitor SAR-020106 (SAR), disrupting homologue recombination, with standard DNA damage inducers (IR, TMZ) and the epigenetic/cytotoxic drug decitabine (5-aza-2′-deoxycitidine, 5-aza-dC). Different in vitro glioblastoma models are monitored to evaluate if the impaired DNA damage repair may chemo/radiosensitize the tumour cells.

**Methods:**

Human *p53*-mutated (*p53*-mut) and -wildtype (*p53*-wt) glioblastoma cell lines (*p53*-mut: LN405, T98G; *p53*-wt: A172, DBTRG) and primary glioblastoma cells (*p53*-mut: P0297; *p53*-wt: P0306) were treated with SAR combined with TMZ, 5-aza-dC, and/or IR and analysed for induction of apoptosis (AnnexinV and sub-G1 assay), cell cycle distribution (nuclear PI staining), DNA damage (alkaline comet or gH2A.X assay), proliferation inhibition (BrdU assay), reproductive survival (clonogenic assay), and potential tumour stem cells (nestin^pos^/GFAP^neg^ fluorescence staining). Potential treatment-induced neurotoxicity was evaluated on nestin-positive neural progenitor cells in a murine entorhinal-hippocampal slice culture model.

**Results:**

SAR showed radiosensitizing effects on the induction of apoptosis and on the reduction of long-term survival in *p53*-mut and *p53*-wt glioblastoma cell lines and primary cells. In *p53*-mut cells, this effect was accompanied by an abrogation of the IR-induced G2/M arrest and an enhancement of IR-induced DNA damage by SAR treatment. Also TMZ and 5-aza-dC acted radioadditively albeit to a lesser extent. The multimodal treatment achieved the most effective reduction of clonogenicity in all tested cell lines and did not affect the ratio of nestin^pos^/GFAP^neg^ cells. No neurotoxic effects were detected when the number of nestin-positive neural progenitor cells remained unchanged after multimodal treatment.

**Conclusion:**

The Chk1 inhibitor SAR-020106 is a potent sensitizer for DNA damage-induced cell death in glioblastoma therapy strongly reducing clonogenicity of tumour cells. Selectively enhanced *p53*-mut cell death may provide stronger responses in tumours defective of non-homologous end joining (NHEJ). Our results suggest that a multimodal therapy involving DNA damage inducers and DNA repair inhibitors might be an effective anti-tumour strategy with a low risk of neurotoxicity.

## Background

Glioblastoma is the most common WHO grade IV brain tumour in adults. The current standard therapy for patients with newly diagnosed glioblastoma includes primary resection, followed by radiotherapy (IR) and adjuvant temozolomide (TMZ) treatment. Despite of this, the median survival remains poor with about 14 months only [1], asking urgently for novel therapy approaches.

Therefore, we tested a new strategy to overcome the high radio- and chemotherapy resistance of glioblastoma cells using the checkpoint kinase 1 (Chk1) inhibitor SAR-020106 (SAR) and the epigenetic modulator and cytotoxic agent decitabine (5-aza-2’-deoxycytidine, 5-aza-dC). The blockage of homologous recombination (HR) by SAR inhibits an important DNA repair mechanism of proliferating cells and should thereby lead to DNA damage accumulation and subsequently to cell death induction. Additionally, decitabine acts as an inhibitor of the de novo methyltransferase 1 (DNMT1) potentially releasing the expression of aberrant promotor-hypermethylated, silenced tumour suppressor genes. Moreover, DNA-incorporated decitabine induces further DNA damage and is therefore expected to synergize with SAR and standard glioblastoma treatment.

Immediately after DNA damage induction, proliferating cells undergo cell cycle arrest for DNA repair before cell division progresses or apoptotic cell death is induced. In mammalian non-cancerous cells usually a G1 phase arrest is activated in a p53-dependent manner and DNA double-strand breaks (DSB) are repaired by non-homologous end joining (NHEJ) [2–5]. However, the *p53* gene is mutated in 30–50% (*p53*-mut) of glioblastomas [6–8]. In the absence of an intact p53 protein DSB are repaired p53-independently preferably by high-fidelity homologous recombination (HR) during S and G2 phases of the cell cycle [9]. Most importantly, elevated HR was recently found to mediate acquired TMZ resistance in recurrent glioblastoma [10]. A critical protein within the activation process of DNA HR is the serine/threonine kinase Chk1 by its interaction with RAD51 [11]. When DNA damage activates ATR (ataxia telangiectasia and Rad3-related protein) Chk1 is activated by phosphorylation [12] and becomes a central regulator of the intra-S and G2/M cell cycle checkpoints (reviewed in [13, 14]). Additional roles of Chk1 in the modulation of cellular response to replication stress are the stabilization of stalled replication forks and the control of replication origin firing (reviewed in [15]).

Inhibition of Chk1 in vitro and in vivo has been shown to have radio- and chemosensitizing effects on cell survival [16] and several Chk1 inhibitors have already been applied in clinical trials (reviewed in [17]). However, most of them failed to improve the therapy efficiency or induced normal tissue toxicity assumedly due to their lack of potency and specificity towards Chk1. SAR is a potent and highly selective ATP-competitive Chk1 inhibitor [18]. It has been shown to enhance the efficacies of other DNA-damaging drugs like irinotecan and gemcitabine in human colon carcinoma xenograft models with minimal toxicities [18, 19]. Furthermore, it sensitized cancer cells to IR-induced DNA damage in vitro and in vivo in head and neck cell carcinoma and colorectal cancer models [18–21].

Decitabine got the FDA approval in 2006 for the treatment of the myelodysplastic syndrome (MDS) and chronic myelomonocytic leukemia (CMML) and EMA approval in 2012 for the treatment of acute myeloid leukemia (AML). Currently, clinical studies are ongoing also for treatment of solid tumours with decitabine in combination with other cytotoxic agents or therapies (https://clinicaltrials.gov/).

Decitabine is an epigenetic DNA-hypomethylating and cytotoxic drug leading to proliferation inhibition and induction of apoptosis by reactivating cancer-related hypermethylation-induced gene silencing of tumour suppressor genes [22, 23] or by induction of DNA damage [24–26]. We have already shown that decitabine massively reduces clonogenic survival and has radioadditive effects in human medulloblastoma cell lines [23]. Although in glioblastoma, the CpG island methylation phenotype (G-CIMP; about 9% of all GBMs) showed a better prognosis, G-CIMP-negative tumours also harbor about 1000 hypermethylated genes involved mainly in regulation of cell development, migration, cell-cell adhesion and transcription factors [27, 28].

In this study, we investigated for the first time the effect of SAR combined with the current standard therapy (IR and TMZ) and decitabine in *p53*-mut and *p53*-wildtype (*p53*-wt) glioblastoma cell lines and primary cells. We analyzed DNA damage, cell cycle phase distribution, proliferation, apoptosis, long-term clonogenic survival, and the number of potential glioblastoma stem cells. Putative toxic effects of this novel treatment approach on non-cancerous normal brain tissue are evaluated on neural progenitor cells using a murine entorhinal-hippocampal slice culture model.

## Material & Methods

### Modulators

Temozolomide (TMZ, trade name Temodal®) and 5-aza-2’-deoxycytidine (5-aza-dC, decitabine, trade name Dacogen®) were purchased from Sigma-Aldrich, SAR-020106 (SAR) from SYNkinase, and CCT244747 from AdipoGen. Stock solutions were prepared as follows: 10 mM 5-aza-dC in PBS (phosphate buffered saline; Biozym; stored at − 20 °C); 100 mM TMZ in DMSO (stored at − 20 °C); 20 mM SAR in DMSO (stored at − 80 °C) or 1 mM in DMSO (stored at 4 °C for max. 1 week); 20 mM CCT244747 in DMSO (stored at − 20 °C). Further work solutions were made in cell culture medium immediately before use, appropriate DMSO controls were implemented.

### Cell lines and cell culture

The human glioblastoma cell lines T98G, DBTRG, and A172 were purchased from the ATCC and LN405 was obtained from the DMSZ. Primary glioblastoma cells P0297 and P0306 were established as described before [29] from primary glioblastoma (IDH wildtype). Patients provided written informed consent according to German laws and in accordance with the 1964 Helsinki declaration and its amendments, as confirmed by the local ethical committee (144/08-ek). *P53* mutation status of primary cells and cell lines was determined by sequencing (Ion AmpliSeq Cancer Hotspot Panel v2, Thermo Fisher Scientific) in the core unit for DNA technologies, Interdisciplinary Centre for Clinical Research Leipzig. The *MGMT* promotor methylation status was determined by pyrosequencing of 5 CpG loci (74–78) (modified after [30]) in the Division of Neuropathology, University of Leipzig. Results are summarized in Fig. 6a. T98G, LN405, and A172 were maintained in DMEM with 4.5 g/l glucose (Biozym) supplemented with 10% FCS (fetal calf serum; Biochrom). DBTRG cells were cultivated in Gibco™RPMI 1640 (Thermo Fisher Scientific) supplemented with 10% FCS, 25 mM HEPES buffer (Lonza), 2.5 g/l (D+) glucose, 0.11 g/l sodium pyruvate (AppliChem), 0.3 g/l L-glutamine, 30 mg/l L-proline, 35 mg/l L-cysteine, 15 mg/l hypoxanthine, 10 mg/l adenine, 1 mg/l thymidine, and 1 mg/l ATP (Sigma-Aldrich). All beforehand mentioned media were supplemented with 100 U/ml penicillin and 100 μg/ml streptomycin (Biochrom). Cells were passaged with trypsin/EDTA. Primary adherent cells were maintained in AmninoMAX-C100 basal medium (Gibco) with 10% AmninoMAX-C100 supplement (Gibco) and passaged using StemPro Accutase (Thermo Fisher Scientific). All cells were cultivated at 37 °C and 5% CO_2_. Vital cells were counted by trypan blue exclusion assay. Tests to detect mycoplasma were performed in three-month intervals using PCR Mycoplasma test kit (AppliChem).

### Animals

Nestin-CFPnuc C57BL/J6 mice [31] were bred in the animal facility of the Faculty of Medicine, University of Leipzig according to European (Council Directive 86/609/EEC) and German guidelines (Tierschutzgesetz) for the welfare of experimental animals as previously described [32]. All experiments had been approved in advance by the local authorities (Landesdirektion Sachsen T12/17).

### Preparation of murine entorhinal–hippocampal slice cultures

Murine organotypic entorhinal–hippocampal slice cultures (OEHSC) were generated from nestin-CFPnuc C57BL/J6 mice on postnatal day (p) 3 to p 6 as initially described by Gahwiler et al. [33] and verified as detailed [32]. Briefly, after decapitation of the mice and preparation of the brains, 350 μm-thick horizontal slices were cut on a vibratome (Leica VT 1000) under sterile conditions. Up to four slices (eight hippocampi) per mouse were collected, the entorhinal–hippocampal formation resected, and transferred onto porous membrane inserts (Millicell PICMORG50, Millipore) in six-well culture plates. The cultivation medium consisted of MEM (Invitrogen) with 25% Hank’s balanced salt solution (Invitrogen), 25% horse serum (Invitrogen), 1% L-glutamine (Sigma), 1% penicillin/streptomycin (Lonza), and 1% glucose (stock solution 45%, AppliChem). Slices were cultivated at 37 °C and 5% CO_2_.

### Irradiation

A 150 kV X-ray machine (DARPAC 150-MC, RayTech) with dose rates of 0.69 Gy/min (75-cm^2^ cell culture flasks), 0.86 Gy/min (6-well plates), or 1.394 Gy/min (96-well plates) was used for IR.

### Apoptosis/cell cycle distribution

Cell death induced by apoptosis was detected by AnnexinV Apoptosis Detection Kit II (BD Pharmingen™) or Annexin-V-FLUOS Staining Kit (Sigma-Aldrich) according to manufacturers’ instructions. Cells were seeded in 6-well plates and allowed to attach for 24 h. One hour after treatment with SAR (0.125 μM, 0.25 μM), cells were irradiated with 8 Gy single dose and treated with 5-aza-dC (0.1 μM, 0.5 μM) or TMZ (50 μM, 100 μM). Cells were harvested by trypsinization 4, 24, and 96 h after IR, washed twice with PBS and stained with Annexin V FITC antibody and propidium iodide. Cell staining was measured by flow cytometry (Beckman Coulter, EPICS XL). Additionally, apoptosis-induced DNA fragmentation was determined in sub-G1 fraction of cell cycle analysis (Nicoletti assay) after propidium iodide staining. Cells were treated and harvested as mentioned above, washed twice with PBS, fixed with 70% ethanol, and stored at − 20 °C overnight. After two washes with PBS cells were incubated with 0.1 mg/ml RNAseA solution (Sigma-Aldrich) at 37 °C for 20 min. Then, 50 μg/ml propidium iodide (Sigma-Aldrich) was added and the solution was incubated at 4 °C for 10 min before DNA content was measured by flow cytometry. Sub-G1 fraction was determined using EXPO32 software (Beckman Coulter).

### Alkaline comet assay

To determine DNA damage in *p53*-mut cells, we used the alkaline comet assay. Cells were seeded in 6-well plates and allowed to attach for 24 h. Then, cells were treated with 0.25 μM SAR and irradiated with 8 Gy single dose 1 h later. Immediately and 24 h after IR, cells were detached using HBSS (Hanks’ balanced salt solution; Gibco) supplemented with 20 mM Na_2_-EDTA and 10% DMSO, washed with PBS, and resuspended in 1% low-gelling temperature agarose (LMPA; Sigma-Aldrich). Then, cells were rapidly spread onto microscope slides pre-coated with a thin layer of 1% normal melting point agarose (NMPA; SeaKem® LE agarose; Biozym) and coated with a thin layer 1% LMPA. Slides were immersed in lysis buffer (2.5 M NaCl, 100 mM Na_2_-EDTA, 10 mM Tris, pH 10.0) at 4 °C overnight. Alkaline denaturation was carried out in pre-chilled electrophorese buffer (300 mM NaOH, 1 mM Na_2_-EDTA, pH > 13) for 60 min, followed by electrophoresis (1.2 V/cm; 370 mA) at 4 °C for 30 min. Then, slides were neutralized by incubation with 0.4 M Tris, pH 7.5 three times for 5 min and stained with 1 μg/ml DAPI. DNA content/tail size of 50 randomly selected nuclei per treatment were measured by image analyses (Comet Assay IV software, Perceptive Instruments ltd.) using a Zeiss AxioLab microscope.

### Immunofluorescence and western blot analyses of DNA damage proteins

To determine the influence of Chk1 inhibition by SAR on proteins involved in DNA damage repair, we used immunofluorescence microscopy and western blot (method see next section) of gH2A.X and phosphorylated replication protein A 32/2 (pRPA). Cells were seeded on 8-well chamber slides and allowed to attach for 24 h. Then, cells were treated with 1 μM SAR-020106 and irradiated with 8 Gy single dose 1 h later. After 1.5, 24 and 72 h, cells were fixed with 2% formaldehyde in PBS for 15 min and immunofluorescence staining was performed as described before [34]. The following primary antibodies were used: mouse anti-phospho-histone H2A.X (Ser139) clone JBW301 (1:100; Millipore); rabbit phospho-RPA32/RPA2 (Ser8) clone E5A2F (1:500; Cell Signaling Technology). Secondary antibodies were as follows: Alexa568 goat anti-mouse IgG F(ab’)2 (1:1000, Invitrogen); Alexa488 goat anti-rabbit IgG F(ab’)2 (1:1000, Invitrogen).

GH2A.X foci were quantified in *p53*-wt primary cells (P0306) in at least 50 cells for each treatment 1 and 24 h after IR. A maximum of 30 gH2AX-foci per nucleus were counted by fluorescence microscopy.

To determine DNA damage-related proteins in T98G cells, we used western blot analysis as already described by Oppermann et al. [35]. In brief, cells were seeded in 100-mm cell culture dishes and allowed to attach for 24 h. Then, cells were treated with 1 μM SAR and 8 Gy single dose irradiation 1 h later. At 0.5, 1.5, 24, and 72 h after irradiation, cells were washed twice with ice-cold PBS, harvested by scraping in 1 ml ice-cold PBS, and the cell suspension was transferred into a 1.5-ml reaction vial. After resuspension in ice-cold RIPA buffer containing phosphatase and protease inhibitors, cell pellets were lysed by sonification. After centrifugation (5500×g; 5 min; 4 °C), the supernatant was transferred into fresh reaction vials. Proteins were immediately frozen at − 80 °C until western blot was performed.

Protein concentration was determined using Pierce™ 660 nm Protein Assay Reagent (Thermo Fisher Scientific) and a BSA (bovine serum albumin) reference standard curve.

SDS-PAGE was performed with a 15% acrylamide gel, 20 μg of protein per lane, and Chameleon Duo protein ladder (Li-COR Biosciences) using a Mini-PROTEAN System (Bio-Rad). After electrophoresis, proteins were transferred to PVDF membranes (Low-Fluorescence Membrane 0.2 μm pore size, Biozym) using a Mini Trans-Blot Cell (Bio-Rad). Then, the membranes were blocked with TBST (Tris-buffered saline with polysorbate 20: 20 mM Tris, 134 mM NaCl, 0.1% Tween 20; pH 7.6) + 2% BSA for 1 h, washed once with TBST for 5 min, and incubated with primary antibodies (mouse anti-phospho-histone H2A.X (Ser139) clone JBW301, Millipore; rabbit phospho-RPA32/RPA2 (Ser8) clone E5A2F, Cell Signaling Technology; rabbit anti-histone H3 clone D1H2, XP® ChIP formulated, Cell Signaling Technology) diluted 1:1000 in TBST for 1 h. Then, membranes were washed three times with TBST for 5 min and incubated with secondary antibodies (red fluorescent IRDye 680RD goat anti-mouse and green fluorescent IRDye 800CW goat anti-Rabbit; both diluted 1:10,000 in TBST; LI-COR Biosciences) for 1 h in the dark. The membranes were washed three times with TBST and once with double-distilled water. All blocking, antibody incubation, and washing steps were performed at room temperature on an orbit shaker. Membranes were scanned using an Odyssey Imaging System (Li-COR, Bad Homburg, Germany).

### Proliferation

Cell proliferation was measured using the colorimetric BrdU cell proliferation ELISA (Sigma-Aldrich) according to the manufacturers’ instructions. For single dose treatment, cells were seeded into 96-well plates and allowed to attach for 24 h. Then, cells were treated with SAR (0.125 μM, 0.25 μM) 1 h before irradiation with 2 or 8 Gy single dose and immediate treatment with 5-aza-dC (0.1 μM, 0.5 μM) or TMZ (50 μM, 100 μM). BrdU solution was added 72 h or 7 d after treatment, 24 h before measurement. For fractionated dose treatment, cells were seeded into cell culture flasks and allowed to attach for 24 h. Then, cells were treated at 7 consecutive days with SAR (0.25, 0.5, 1 μM) and IR (2.2/3.4 Gy per fraction) 1 h later (see treatment schedule Fig. 6c). Five days after treatment, cells were seeded in BrdU-containing medium into 96-well plates and measured 24 h later.

### Clonogenic survival

To examine long-term survival of clonogenic cells after single drug treatment, cells were seeded in 6-well plates at three different cell densities in duplicates, allowed to attach overnight, and treated with different drug concentrations at day 1–3 and 6–9 (7 fractions, see Fig. 6c). Irradiation and immediate TMZ or 5-aza-dC treatment was performed 1 h after SAR administration. On day 14, fixation and staining of colonies was performed as detailed below.

For clonogenic assays after multimodal treatment, the setting had to be adapted to the higher cell death rate compared to single treatments: Cells were seeded in 75-cm^2^ cell culture flasks and allowed to attach overnight. Medium was changed daily and fractionated treatment was executed (see Fig. 6c). At day 14, vital cells were counted using trypan blue exclusion test and seeded for clonogenic assay at three different cell densities in duplicates in 6-well cell culture plates. Ten to 17 days later (dependent on cell line), colonies were washed with PBS, fixed with ice-cold ethanol/acetone (1/1, V/V) for 10 min, stained with Giemsa (Dr. K. Hollborn & Söhne GmbH & Co. KG) solution (1/1, V/V with distilled water) for 5 min, and washed with distilled water. Colonies with > 50 cells were counted indicating the plating efficiency (PE). The ratio between PE of treated cells and PE of untreated cells represented the surviving fraction (SF) of clonogenic cells. The overall clonogenic survival (OSF) was calculated from the relative number of vital cells at day 14 multiplied with the SF (only multimodal treatment).

### Immunofluorescence microscopy of potential tumour stem cells

For nestin and GFAP staining, cells were seeded on 8-well chamber slides 5 d after fractionated treatment (7 fractions, see Fig. 6c) and allowed to attach for 24 h. Then, cells were washed in PBS, fixed in ethanol/acetone (1/1, V/V) for 10 min, permeabilized with 0.5% Triton-X100 for 5 min, and immersed in PBS with 10% normal goat serum and 0.25% Triton-X-100 for 30 min to block unspecific binding. Slides were then incubated with the primary antibody: mouse IgG1 anti-human nestin clone10C2 (Millipore, Cat# MAB5326) 1: 200; rabbit Ig anti-human GFAP (DAKO, Cat# Z0334) 1: 500 in PBS with 2% normal goat serum and 0.25% Triton-X100 at 4 °C overnight. After three washes with PBS, the slides were incubated with the secondary antibodies: goat anti-mouse IgG F(ab’)2-Alexa488 or goat anti-rabbit IgG F(ab’)2-Alexa568 (Invitrogen) 1: 1000 in PBS with 2% normal goat serum and 0.25% Triton-X100 at IR for 1 h. Nuclei were counterstained with DAPI (4′6-diamidino-2-phenylindole-dilactate 10 mg, 1: 10,000; Invitrogen) for 5 min and slides were mounted in Mowiol 4–88/DABCO (Roth, Sigma-Aldrich). For all stainings, specific IgG isotype controls (nestin: mouse IgG1, 1: 100, Millipore; GFAP: rabbit Ig, 1: 2500; DAKO) were applied.

### Live imaging analyses of neural progenitor cells

To assess the neurotoxic potential of the multimodal treatment, murine entorhinal–hippocampal slice cultures were fractionated treated (7 fractions, see Fig. 6c) and the nestin expression, characteristic for neural progenitor cells, was visualized after 9 and 16 days using an Olympus BX51 confocal fluorescence microscope at 458 nm excitation and quantified using ImageJ and the Plugin Cell Counter (http://imagej.nih.gov/ij/) as previously described [32].

### Statistics

Statistical data analyses were, if not otherwise noted, performed using the parametric, two-way, and paired Student’s *t*-test with Microsoft Excel 2003 software.

Statistical analyses of clonogenic survival data were performed using non-parametric Mann-Whitney test with SPSS statistic software version 24.

*P*-values ≤0.05 (*;^#^) and ≤ 0.01 (**;^##^) were considered as statistically significant and *p*-values ≤0.001 (***;^###^) as highly statistically significant.

## Results

**Fig. 1:**
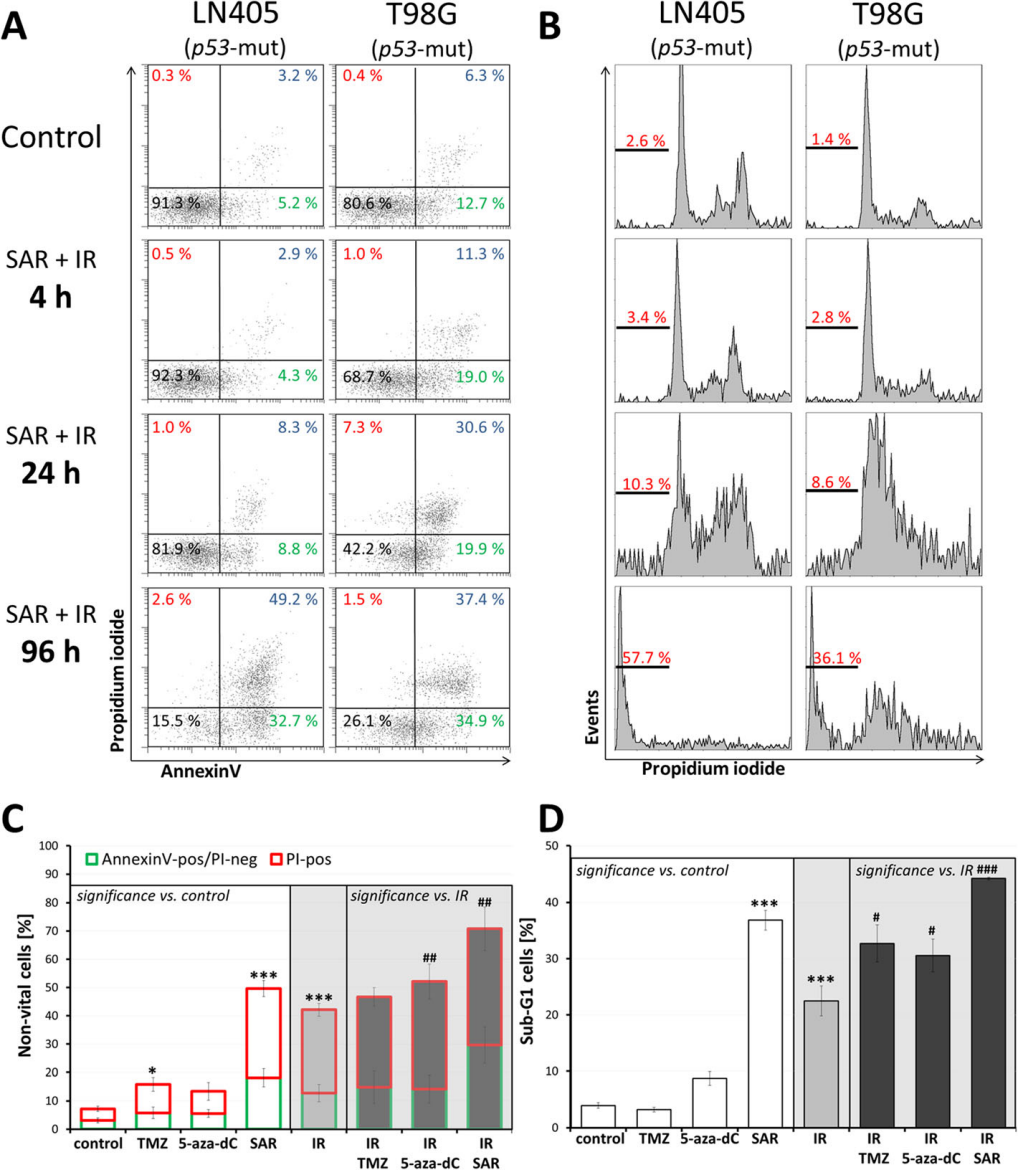
Induction of apoptosis/cell death in LN405 and T98G glioblastoma cell lines. **a** Dot plot analysis after AnnexinV assay and **b** histograms presenting apoptotic DNA fragments (sub-G1 fraction) after cell cycle analysis (Nicoletti assay) 4, 24, and 96 h after combined treatment with 0.25 μM SAR and 8 Gy single dose irradiation. One representative experiment is figured in **a** and **b**. **c**, **d** Joined analyses of apoptosis induction (**c** AnnexinV/propidium iodide staining; **d** sub-G1 cell fraction) in both cell lines 96 h after single dose treatment with 0.25 μM SAR, 50 μM TMZ, 0.1 μM 5-aza-dC, and/or 8 Gy IR. Data are weighted means ± medium SEMs from three independent experiments of each cell line. Statistical significance is given compared to untreated, non-irradiated control and indicated by asterisks (*, *p* ≤ 0.05; ***, *p* ≤ 0.001), compared to IR alone by hashtags (#, *p* ≤ 0.05; ##, *p* ≤ 0.01; ###, *p* ≤ 0.001). All irradiated groups are significantly different to the control group (*p* ≤ 0.01) and therefore no further labeling by asterisks is shown

**Fig. 2:**
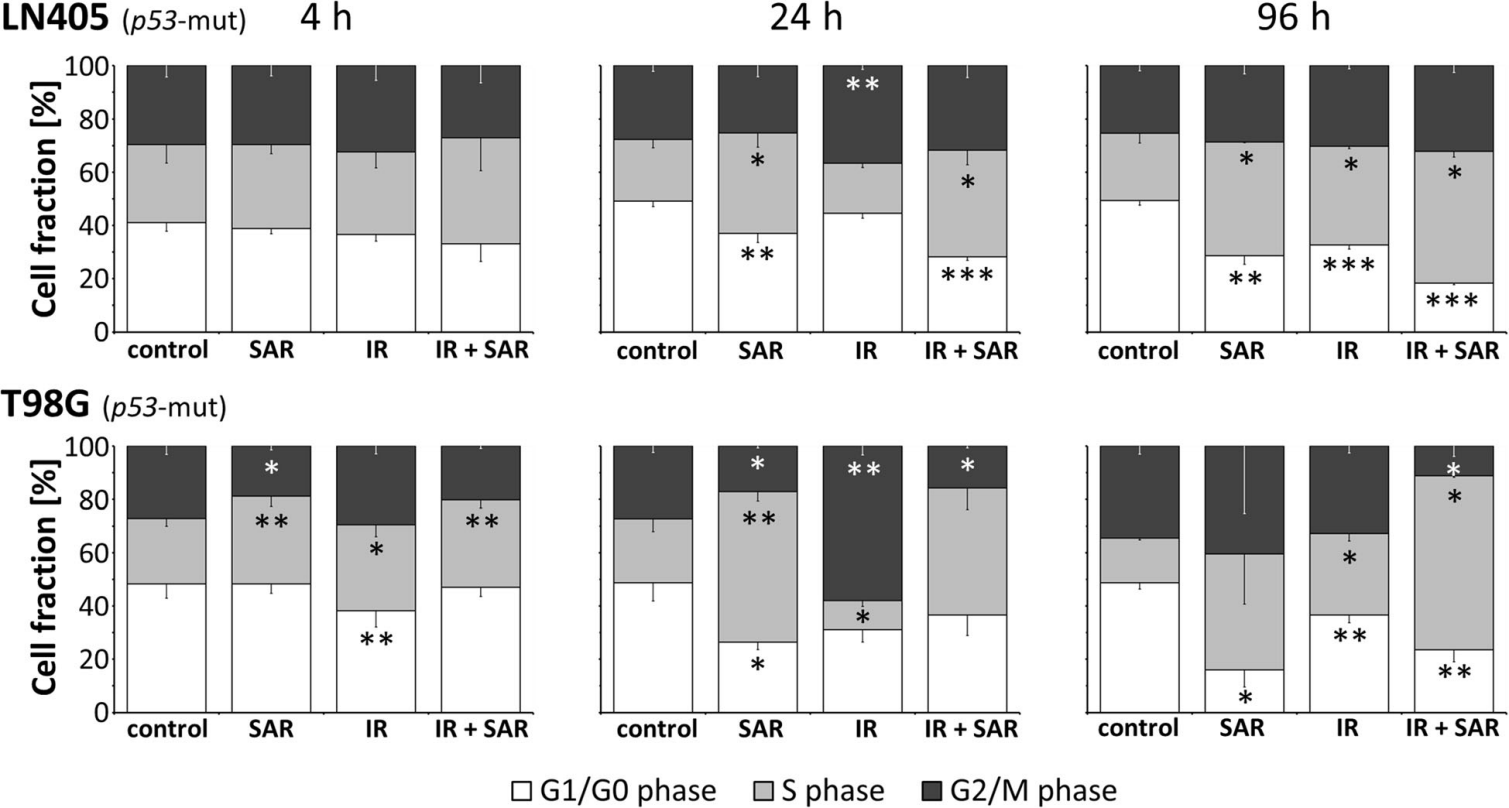
Cell cycle distribution of LN405 and T98G glioblastoma cell lines 4, 24 and 96 h after treatment with 0.25 μM SAR and/or 8 Gy irradiation. Data are means ± SEM from 3 independent experiments. Statistical significance compared to control is indicated by asterisks (*, *p* ≤ 0.05; **, *p* ≤ 0.01; ***, *p* ≤ 0.001)

**Fig. 3:**
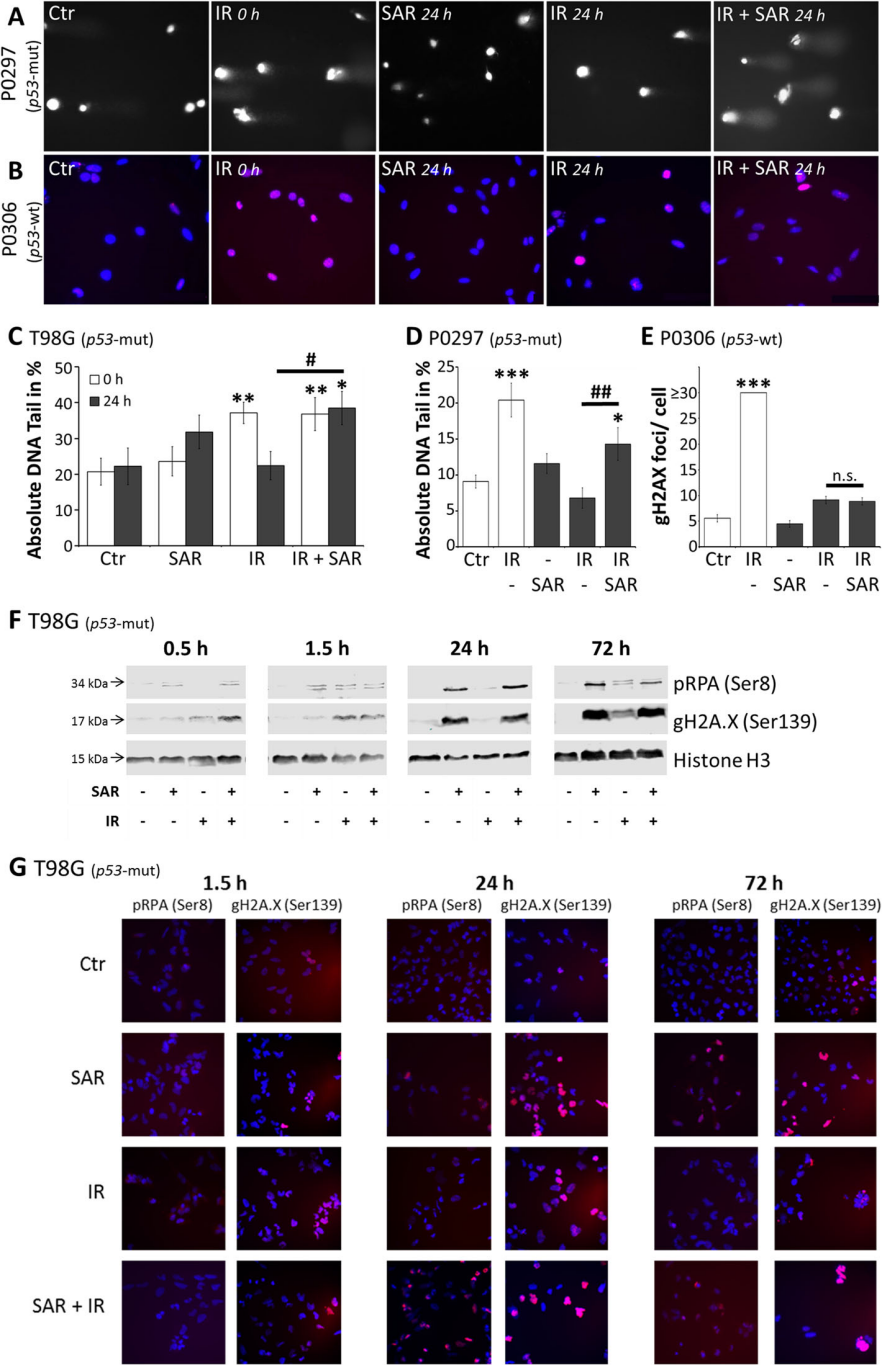
Induction of DNA damage. **a**, **b** Representative photographs after **a** alkaline comet assay or **b** gH2AX assay (blue, DAPI nuclear stain; red, gH2A.X protein) of primary glioblastoma cells. **c** Relative comet tail DNA of T98G cells after treatment with 0.25 μM SAR and/or irradiation with 8 Gy. **d** Relative comet tail DNA of P0297 and **e** number of gH2AX foci of P0306 primary glioma cells after treatment with 1 μM SAR and/or irradiation with 8 Gy. Analyses were conducted immediately (white bars) or 24 h (black bars) after IR. Data are means ± SEM from one experiment with at least 50 analyzed cell nuclei. Statistical significance compared to control is indicated by asterisks (*, *p* ≤ 0.05; **, *p* ≤ 0.01; ***, *p* ≤ 0.001), compared to irradiated group by hashtags (#, *p* ≤ 0.05; ##, *p* ≤ 0.01). **f** Western blot analyses of phosphorylated RPA (Ser8) and gH2A.X of T98G cells after treatment with 1 μM SAR and/or irradiation with 8 Gy. Histone H3 protein was used as loading control. **g** Representative photographs after immunofluorescence staining of phosphorylated RPA (Ser 8) and gH2A.X in T98G cells after treatment with 1 μM SAR and/or irradiation with 8 Gy

**Fig. 4:**
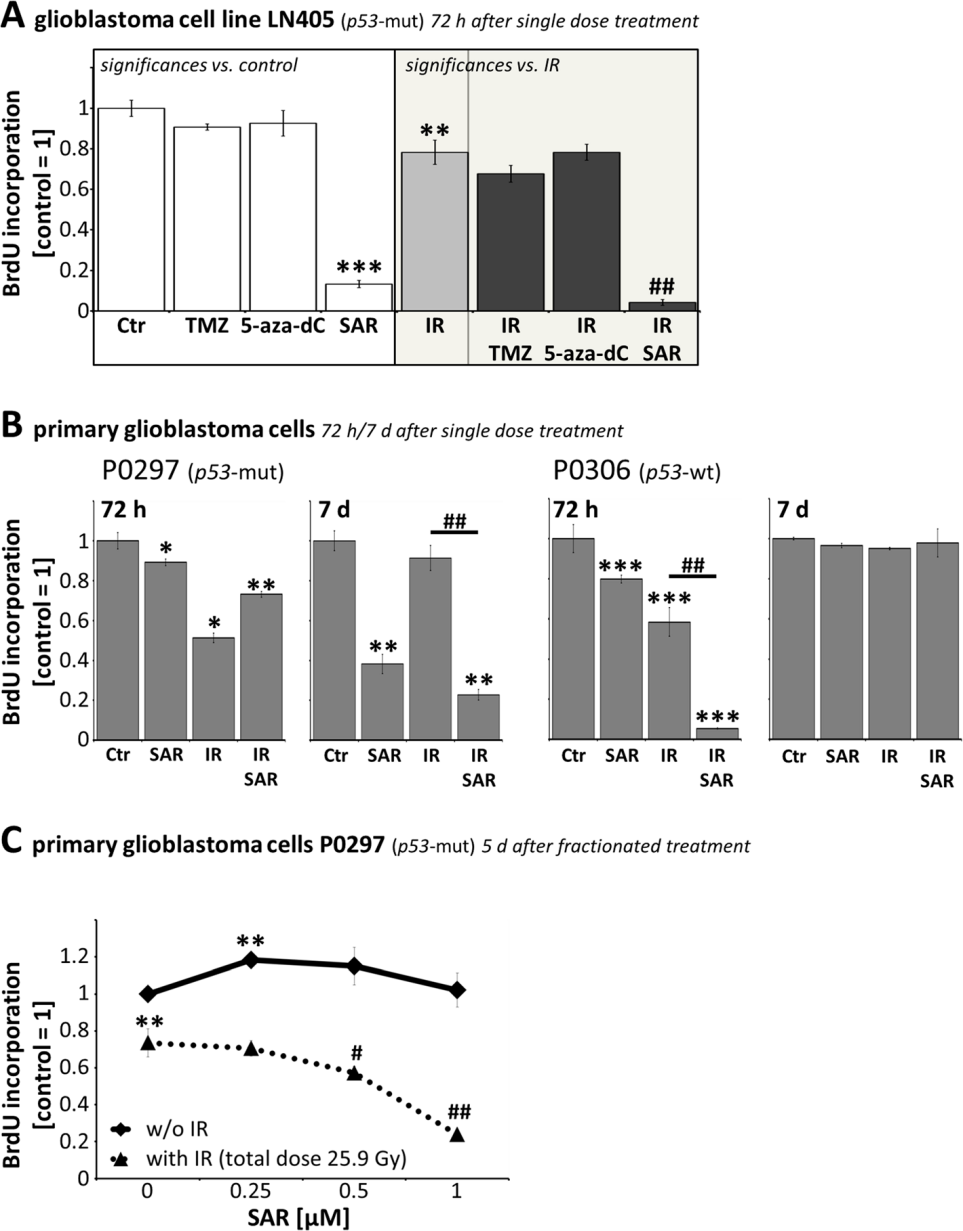
Proliferation of glioblastoma cells. **a** BrdU incorporation in LN405 72 h after single dose treatment with 0.25 μM SAR, 50 μM TMZ, 0.1 μM 5-aza-dC and/or 8Gy IR. **b** BrdU incorporation in primary glioblastoma cells 72 h and 7 d after single dose treatment with 0.25 μM SAR and/or 2 Gy IR. **a**, **b** Data are weighted means ± medium SEM from three independent experiments (except P0306 7 d post IR, *n* = 2). **c** BrdU incorporation 5 d after fractionated treatment with different SAR concentrations and/or 25.9 Gy total IR dose (7 × 3.4 Gy). Data are means ± SEM from one experiment performed at least in triplicates. **a**-**c** Statistical significance compared to untreated, non-irradiated control is indicated by asterisks (*, *p* ≤ 0.05; **, *p* ≤ 0.01; ***, *p* ≤ 0.001), compared to IR alone by hashtags (#, *p* ≤ 0.05; ##, *p* ≤ 0.01). In **a**, all irradiated groups differed significantly compared to untreated, non-irradiated control (*p* ≤ 0.01) and therefore no further labeling by asterisks is shown

**Fig. 5:**
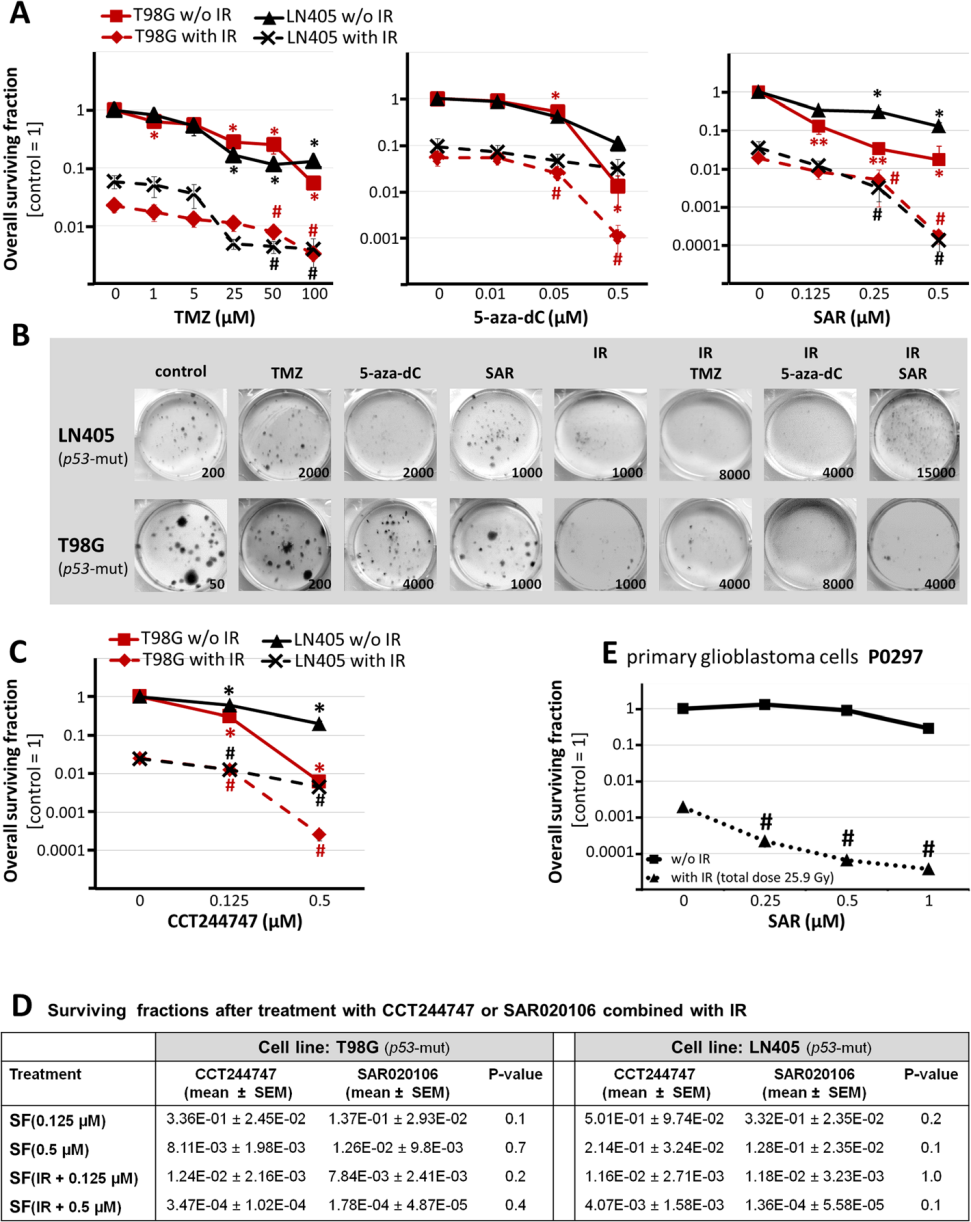
**a** Clonogenic survival of *p53*-mutant glioblastoma cell lines after fractionated single drug treatment with SAR, 5-aza-dC, and TMZ with or without irradiation (7 × 2.2 Gy, total dose 15.4 Gy). **b** Representative photographs of stained colonies after clonogenic assay. Concentrations were as follows: 0.25 μM SAR, 0.5 μM 5-aza-dC, 50 μM TMZ. Numbers indicate seeded cells per well. **c** Clonogenic survival after fractionated single drug treatment of *p53*-mutant glioblastoma cell lines with CCT244747. **a**, **c** Data are means ± SEM from 3 independent experiments. **d** Surviving fractions (means ± SEM) of *p53*-mutant glioblastoma cell lines after treatment with CCT244747 or SAR020106 and/or IR. **e** Overall surviving fraction of *p53*-mutant, primary P0297 cells. Data are means ± SEM from one experiment in sextuplicates. **a**, **c**, **e** Statistical significance compared to untreated, unirradiated control is calculated by Mann-Whitney test and indicated by asterisks (*, *p* ≤ 0.05; **, *p* ≤ 0.01). All irradiated groups are significantly different compared to untreated, non-irradiated control (*p* ≤ 0.05) and therefore no further labeling is shown. Statistical significance compared to untreated, irradiated control is indicated by hashtag (#, *p* ≤ 0.05)

**Fig. 6:**
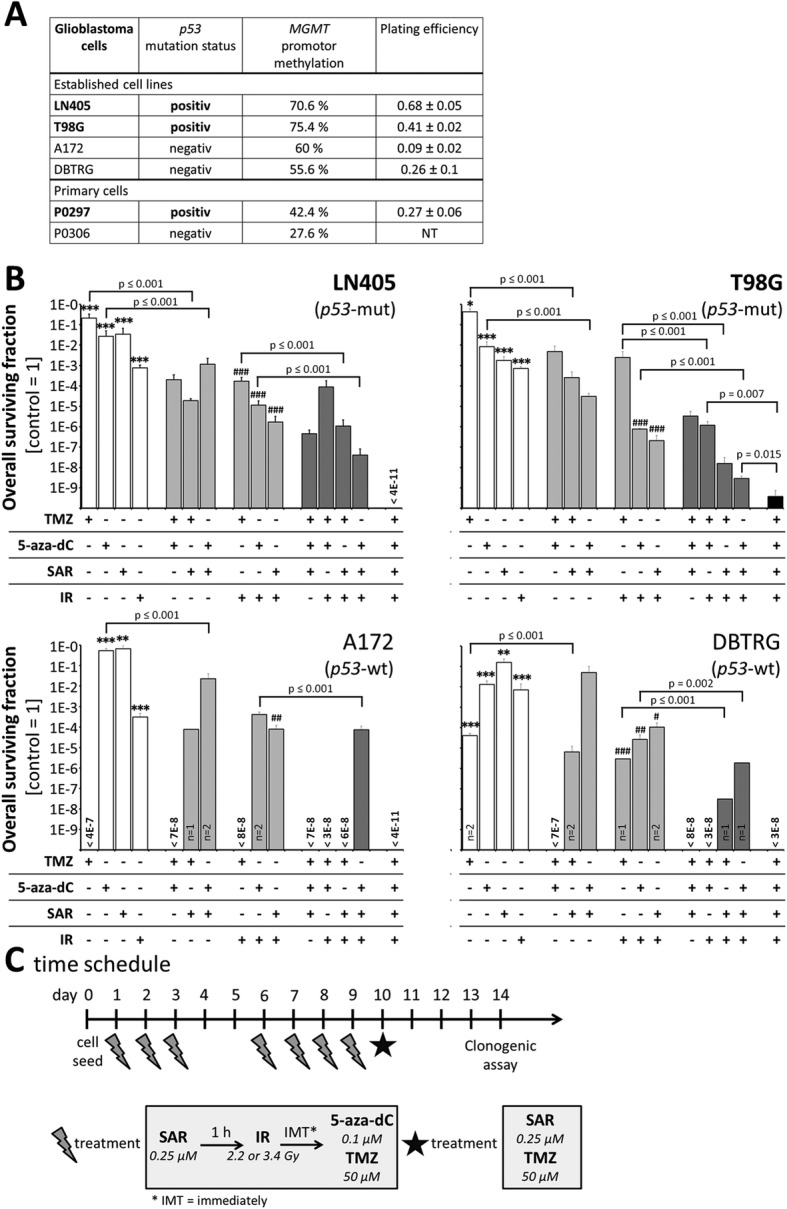
Overall clonogenic survival after fractionated multimodal treatment. **a** Molecular features (*p53* mutation status, *MGMT* promotor methylation and gene expression) and determined plating efficiencies of glioblastoma cell lines and primary cells. NT means not tested. Data are means ± SEM from 3 independent experiments. **b** Overall surviving fractions of established cell lines after fractionated (7x), multimodal treatment with 0.25 μM SAR, 50 μM TMZ, 0.1 μM 5-aza-dC, and 2.2 Gy IR (total dose 15.4 Gy). Data are means ± SEM from 3 independent experiments (if not otherwise noted at the bottom of the bar) in sextuplicates. Significance of single treatments compared to untreated, non-irradiated control is indicated by asterisks (**, *p* ≤ 0.01; ***, *p* ≤ 0.001). Statistical significance of combined treatments with single drug plus IR compared to IR control is indicated by hashtags (##, *p* ≤ 0.01; ###, *p* ≤ 0.001). Selected significances of comparitive analyses are shown. **c** Time schedule of the fractionated multimodal treatment

**Fig. 7:**
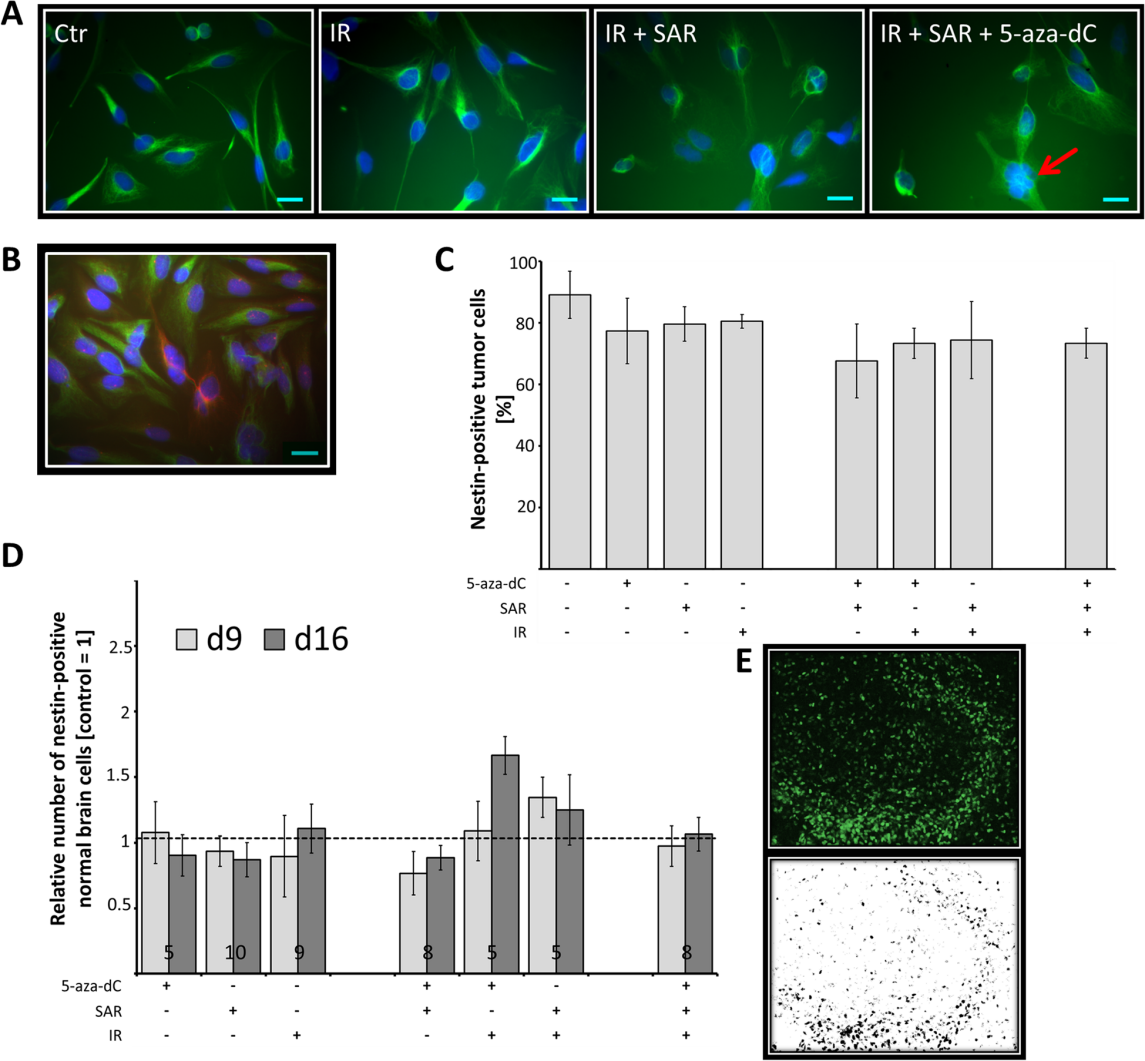
Nestin-positive cells. **a**-**c** Potential tumour stem cell population of primary glioblastoma cells P0297 and **d**, **e**. Normal neural progenitor cells in murine hippocampal slice cultures both fractionated (7x) treated with 0.1 μM 5-aza-dC, 0.25 μM SAR and 2.2 Gy (total dose 15.4 Gy). **a** Representative photographs of nestin staining (green) and DAPI nuclear stain (blue). **b** Double-stained P0297 cells: nestin (green); GFAP (red); DAPI nuclear counterstain (blue). Scale bar of A and B = 20 μm. **c** Absolute amount of nestin-positive glioblastoma cells. Data are means ± SEM from one experiment with three independent counts of at least 100 cells in total. No significances were detected. **d** Relative amount of nestin-positive neural progenitor cells within the dentate gyrus normalized to untreated control. Data are means ± SEM, ciphers at the bar bottom indicate the number of animals used. No significances were detected. **E** Representative photograph of the dentate gyrus (nestin-fluorescent cells, green) and the corresponding analysis by ImageJ (counted cells, black)

Multimodal treatment mechanisms were analysed on two human glioblastoma cell lines (LN405 and T98G) to analyse effects of SAR specifically in *p53*-mut tumours (Figs. 1, 2, 3, 4, 5 and 6). A172 and DBRTG cell lines were additionally implemented for comparative analysis of long-term survival in *p53*-wt glioblastomas (Fig. 6). In addition, key experiments (DSB induction, proliferation effects, clonogenicity, stem cell ratio) were verified on primary glioblastoma cells obtained from one *p53*-mut and one *p53*-wt patient (Figs. 3, 4, 5, 7)

### Mechanisms of cell death induction in p53-mut cell lines by multimodal treatments

The annexin localization in the plasma membrane (Annexin V assay), the fractionated DNA content (sub-G1 Nicoletti assay) and the membrane leakage (PI staining) were measured to determine cell death induction 4, 24, and 96 h after IR. The *p53*-mut glioblastoma cell lines LN405 and T98G showed similar results with strongest effects after 96 h and therefore were jointly analysed at this time point. Whereas TMZ and 5-aza-dC had only marginal effects, SAR (0.25 μM) and IR (8 Gy) induced apoptosis enhancing control levels by 15 and 10% AnnexinV^pos^/PI^neg^ cells and by 35 and 18% sub-G1 cells. Also the number of non-vital cells (AnnexinV^pos^ and PI^pos^) increased by 43 and 35%. In combination with IR, all mediators showed significant radioadditive effects, most pronounced after combined IR/SAR treatment, enhancing control levels by 27% AnnexinV^pos^/PI^neg^ cells, 64% non-vital cells, and by 40% sub-G1 cells (Fig. 1c, d)

### Changes of cell cycle distribution in p53-mut cell lines by SAR and IR

Cell cycle analysis of *p53*-mut glioblastoma cell lines LN405 and T98G was performed by flow-cytometric DNA content measurement 4, 24, and 96 h after IR

After treatment with TMZ (50 and 100 μM) and 5-aza-dC (0.1 and 0.5 μM) alone or in combination with IR, no significant changes of cell cycle distribution were observed (data not shown). In contrast, SAR induced an accumulation of cells in S phase in both cell lines (by 15% in LN405 and 32% in T98G at 24 h), accompanied by a reduction of cells in G2/M phase in T98G at 4 and 24 h (10%) compared to untreated control. IR led to an enhancement of G2/M cells at 24 h (by 9% in LN405 and 31% in T98G), which was reversed to control level at 96 h. SAR abrogated the IR-induced G2/M arrest (Fig. 2)

### Enhanced induction of DNA damage by IR and SAR in p53-mut cells

DNA double-strand break (DSB) induction was quantified by gH2A.X assay in *p53*-wt primary cells, whereas in *p53*-mut cells the initial foci number was already > 30 and DNA damage was therefore measured by alkaline comet assay.

In *p53*-mut T98G and primary P0297 cells, SAR alone did not enhance the amount of comet tail DNA significantly. Immediately after IR a 1.8 and a 2.2-fold increase of comet tail DNA was detected, which was reversed to control level 24 h after IR. The combination of SAR and IR did not change the comet tail DNA immediately after IR but showed a 1.7 (T98G) and 2.1-fold (P0297) enhancement of residual comet tail DNA compared to IR alone 24 h after IR (Fig. 3a, c, d)

In contrast, in *p53*-wt cells (primary P0306), the gH2A.X assay revealed no effect of SAR on IR-induced residual DNA damage repair (Fig. 3b, e)

The results of the gH2A.X assay were confirmed by western blot analysis showing enrichment of gH2A.X and pRPA DNA damage/repair proteins 0.5 and 1.5 h after IR or IR + SAR in *p53*-mut T98G cells. At later time points (24 h and 72 h), SAR increased the IR-induced amount of gH2A.X and pRPA proteins. At high concentration (1 μM), SAR itself also led to an accumulation of gH2A.X and pRPA proteins (Fig. 3f). Similarily, immunofluorescence staining of nuclear gH2A.X and pRPA proteins was enhanced after treatment with IR and/or cotreatment with SAR, reaching highest levels after 24 and 72 h (Fig. 3g)

### Differential effect of SAR on IR-induced proliferation stop

Proliferation was determined by colorimetric BrdU incorporation assay in adherent glioblastoma cell lines and primary cells (Fig. 4a-c)

Treatment of *p53*-mut LN405 cells with TMZ (50 μM) or 5-aza-dC (0.1 μM) did not affect the proliferation and did not enhance the effect of IR (Fig. 4a). SAR alone (0.25 μM) and IR alone (8 Gy) reduced the proliferation to 0.13 ± 0.02 and 0.78 ± 0.06 at 72 h, compared to control level 1.0 ± 0.04. Their combination reduced the proliferation further to 0.04 ± 0.01

Also in primary *p53*-mut (P0297) and *p53*-wt (P0306) cells, similar anti-proliferative effects of SAR alone, of IR (2 Gy) alone, and of their combinations were found 72 h after IR (Fig. 4b). In P0297 cells a more pronounced effect of SAR was observed at 7 d versus 72 h, reducing the proliferation to 0.38 ± 0.05 (SAR alone) and to 0.23 ± 0.03 (SAR combined with IR), whereas the proliferation in irradiated-only cells nearly returned to control levels. In contrast, in P0306 the strongest anti-proliferative effect was determined at 72 h with a reduction to 0.8 ± 0.02 (SAR alone), 0.58 ± 0.07 (IR alone), and 0.05 ± 0.01 (SAR and IR). At 7 d, the number of proliferating P0306 cells had already returned to control levels

Fractionated treatment (schedule see Fig. 6d) combining SAR (0.25 to 1.0 μM) and IR (7 × 3.4 Gy, total dose 25.9 Gy) in primary *p53*-mut (P0297) cells showed an additive effect of SAR on the IR-induced proliferation inhibition at SAR concentrations ≥ 0.5 μM (Fig. 4c)

### Concentration-dependent reduction of clonogenic survival after single drug treatments

The reproductive long-term survival of tumour cells after treatment was examined by clonogenic assay experiments in T98G and LN405 cells. Fractionated single drug treatments with and without IR were conducted to evaluate the concentrations to be used in combinatory experiments, to prove the dose dependency, and therefore the specificity of the single treatments. When human glioblastoma patients are treated with TMZ, it is applied 1 h before IR. To test whether this schedule is also the most effective one in vitro, preliminary experiments were conducted, where we treated LN405 and T98G cells 1 h before IR or immediately after IR. In the latter schedule a significantly higher clonogenic cell death induction was observed, therefore, TMZ was applied immediately after IR in all combination experiments. Significant decreases of overall surviving fractions (OSFs) were found in both cell lines at concentrations of TMZ ≥ 25 μM; 5-aza-dC ≥ 0.05 μM (T98 G only), and SAR ≥ 0.25 μM. Fractionated IR (7 × 2.2 Gy, total dose 15.4 Gy) reduced the OSF to 0.06 ± 0.02 in LN405 and 0.03 ± 0.01 in T98G (Fig. 5a), and diminished the diameter of colonies. Also, more vital single cells were seen after IR, independently from additional drug treatment (Fig. 5b). TMZ, 5-aza-dC, or SAR had significant radioadditive effects on OSF, at TMZ ≥ 50 μM, 5-aza-dC ≥ 0.5 μM, and SAR ≥ 0.25 μM (Fig. 5a)

Additionally, the anti-clonogenic effect of the orally bioavailable Chk1 inhibitor CCT244747, most closely related to SAR [36], was evaluated in T98G and LN405 cell lines. Concentration-dependent reduction of OSF and radiosensitization by CCT244747 was found to be very similar to SAR, at ≥ 0.125 μM (Fig. 5c, d)

To determine whether SAR has also an effect on *p53*-Mut primary glioblastoma cells, different SAR concentrations combined with IR (7 × 3.4 Gy, total dose 25.9 Gy) were tested in P0297 cells (Fig. 5e) using the same fractionated treatment schedule but with an adapted, higher IR dose (Fig. 6c). Thereby, SAR itself showed no significant toxicity but revealed significant radioadditive effects at concentrations ≥ 0.25 μM. The anti-clonogenic impact of IR, and IR + SAR was less in primary cells than in glioblastoma cell lines. For example, the OSF of SAR-treated, irradiated LN405 (OSF _[0.25 μM SAR + 15.4 Gy]_) was 1.7E-06 ± 1.6E-06, whereas the OSF of primary P0297 (OSF_[0.25 μM SAR + 25.9 Gy]_) was 2.2E-04 ± 7.3E-05

### Clonogenic survival after multimodal treatments in p53-mut and -wt cell lines

For the multimodal treatment regime, see Fig. 6c, we decided from the data above (Fig. 5a) to use the following drug concentrations which induced significant but submaximal effects on OSF: 50 μM TMZ, 0.1 μM 5-aza-dC, 0.25 μM SAR. To examine, whether effects are p53-dependent, *p53*-mut (LN405, T98G) and *p53*-wt glioblastoma cell lines (A172, DBTRG) were employed. The treatment with TMZ as single agent as well as in combinations revealed stronger effects in *p53*-wt than in *p53*-mut cell lines, which all exhibited a strong *MGMT* promoter methylation (molecular features see Fig. 6a). 5-Aza-dC diminished the clonogenic survival in all tested cell lines to similar extends. Both, TMZ and 5-aza-dC acted radioadditively. After treatment with SAR, a stronger decrease of clonogenicity was observed in *p53*-mut than in *p53*-wt cell lines, although SAR induced significant additive effects on irradiated cells and also on combined-treated cells receiving IR/TMZ or IR/5-aza-dC in both cells. All multidrug treatments without IR showed lesser effects on clonogenic cell death than those involving IR, whereby SAR enhanced TMZ and 5-aza-dC effects also without IR. The multimodal treatment composed of TMZ, 5-aza-dC, SAR, and IR was most effective, e. g. leading to OSFs between < 4E-11 (LN405, no colonies were grown) and 3.7E-10 ± 3.7E-10 (T98G) (Fig. 6b)

### No effect of multimodal treatment on tumour progenitor cell ratio

To examine the amount of potential tumour progenitor cells (pTPC), nestin/GFAP immunofluorescence double-staining was performed after multimodal treatment of primary *p53*-mut glioblastoma cells (P0297). Quantification revealed that 98% of P0297 cells were nestin-positive/GFAP-negative pTPCs, independent of any treatment (Fig. 7a, c). Only 2% showed GFAP positive staining (Fig. 7b) indicating differentiation. The formation of multinucleated cells was observed after combined treatment with 5-aza-dC, SAR, and IR (Fig. 7a, red arrow)

### Evaluation of potential neurotoxic effects on the number of nestin-positive neural progenitor cells

Live imaging microscopy revealed no significant change of nestin-positive neural progenitor cells in a murine hippocampal tissue slice model 9 and 16 days after multimodal treatment. Toxic effects on the number of normal nestin-positive neural progenitor cells within the dentate gyrus (Fig. 7d, e) could not be observed in any treatment group

## Discussion

Anticancer treatment by irradiation- or drug-induced DNA damage is limited by the DNA repair capacity of tumour cells. The DNA damage-induced G1/S arrest is accompanied by the non-homologous end joining (NHEJ) repair pathway which is inactive in about 50% of glioblastomas due to aberrant p53 signalling. The inhibition of the alternative p53-independent G2/M DNA repair checkpoint by Chk1 inhibitors may lead to the accumulation of DNA damage resulting in a specific enhancement of tumour cell kill. To exploit this promising strategy, we investigated in this study for the first time antitumour effects after combined application of the Chk1 inhibitor SAR-020106 together with irradiation, temozolomide and decitabine in *p53*-wildtype and -mutated human glioblastoma cells in a clinical relevant, fractionated setting.

Analysis of cell death induction in two *p53*-mut glioblastoma cell lines (LN405, T98G) revealed that IR induces apoptotic and non-apoptotic cell death. This is going along with the notion that IR-induced DNA damage mainly results in cell death by mitotic catastrophe or replicative senescence (reviewed in [13, 37]). Addition of the S and G2/M checkpoint inhibitor SAR led to a significant enhancement even of apoptotic cell death (AnnexinV^pos^/PI^neg^; Fig. 1**)**. This is in line with findings of Borst et al. [21] for SAR and also for other Chk1 inhibitors [17] and may indicate the induction of p53-independent apoptotic pathways e.g. via activation of p73 through Chk2 [38, 39]. Also, given as single mediator, SAR strongly induced cell death in these *p53*-mut cells (Fig. 1). This might be explained by the high baseline amount of DSB observed in gH2A.X assays, presumably caused by inefficient NHEJ and further accumulation of DSB after SAR administration through its abrogation of G2/M arrest (Fig. 2) and homologue recombination (HR) DNA repair [18, 21]. Additionally, the prolongation of the S cell cycle phase by SAR (Fig. 2) indicates SAR-induced DNA synthesis problems, in line with its known inhibition of Chk1, and the role of Chk1 during S phase activities (reviewed in [15]). In comet assays, we revealed that only in SAR-treated *p53*-mut cells IR-induced DSB remained for at least 24 h (Fig. 3a-d) going along with its function as DNA repair inhibitor [11]. Syljuasen et al. [40] suggested that the inhibition of Chk1 in S phase cells increased the binding of pRPA to single-stranded DNA leading to genomic instability and DSB. Additionally, the hyperphosporylation of RPA indicated by phosphorylation at S4/S8 (Fig. 3f) was shown to go along with DSB generated from the collapse of replication forks after treatment with DNA-damaging agents, e.g. Chk1 inhibitors, stalling DNA replication [41]. The enhanced accumulation of the DNA damage/repair proteins pRPA and gH2A.X observed here by western blot and immunofluorescence supports the notion that DNA damage is induced, especially DSB, and that DNA repair in response of combined IR and SAR treatment is delayed. At high concentrations, also SAR alone seems to inhibit the repair most probably of spontaneous DSB in *p53*-mut cells (Fig. 3f,g), which is in line with our cell death (Fig. 1), cell cycle (Fig. 2), and proliferation results (Fig. 4**a**).

In the *p53*-mut glioblastoma cell line LN405 we observed a significant reduction of BrdU incorporation by SAR and by IR after 72 h which was pronounced after combination of both (Fig. 4a), probably as a result of cell death as shown in the apoptosis assay (Fig. 1). This result was verified in primary glioblastoma cells which showed a lower proliferation rate, resulting in a postponed response. Here, SAR moderately reduced the BrdU incorporation after 72 h and partly reversed the IR-induced reduction of proliferation in *p53*-mut cells (presumably by overwriting the IR-induced G2/M arrest shown in Fig. 2). In contrast, SAR enhanced the IR effect on BrdU incorporation in *p53*-wt cells at 72 h, where the unrepaired DSB may lead immediately to apoptosis, explaining the lack of effects after 7 days. In the *p53*-mut cells however, only at the later time point (7 days) SAR pronounced the IR-induced reduction of BrdU incorporation, going along with the hypothesis, that accumulation of DSB after combination of SAR and IR may lead to delayed cell death by mitotic catastrophe (Fig. 4b) (reviewed in [13]). These results were also confirmed in a fractionated and therefore more clinical relevant setting in *p53*-mut primary GBM cells (Fig. 4c).

Clonogenic survival experiments in the fractionated setting underlined the above findings, showing accelerated reproductive cell death by SAR in all four glioblastoma cell lines after IR, 5-aza-dC, or TMZ treatment with slightly less response in *p53*-wt cell lines. Interestingly, Bao et al. reported an enhanced Chk1/2 activity especially in glioma stem cells which are thought to promote radioresistance, underlining the relevance and selectivity of DNA checkpoints as therapeutic targets [42]. We could also confirm here, that decitabine can sensitize glioblastoma cells towards TMZ and IR. This might be induced by the observed gene body hypomethylation and reexpression of *MGMT*, a gene coding for a DNA mismatch repair protein essential for TMZ-induced DNA damage repair, and by the enhancement of residual DNA damage after IR [43, 44]. Treatments including TMZ induced stronger cell death in *p53*-wt compared to *p53*-mut cell lines with similar *MGMT* promoter methylation status (55.6–75%) which is in accordance with the reported role of p53 in cancer drug resistance [45]. However, strongest anti-clonogenic effects were seen after triple combination of SAR with IR, 5-aza-dC, and TMZ again in both, *p53*-mut and *p53*-wt glioblastoma cell lines (Fig. 6) supporting the multimodal treatment approach.

The significance of the *p53* mutation status regarding the sensitivity of tumour cells to Chk1 inhibitors like SAR varies in the literature (overview in [13]). Especially in *p53*-wt cells aberrations of proteins downstream of p53 may also lead to abnormal G1/S checkpoint control resulting in similar effects as seen in *p53*-mut cells. However, in our case the low number of unrepaired spontaneous DSB (Fig. 3b, e; gH2A.X assay) indicates a functional NHEJ in the *p53*-wt primary cells (P0306). Also crossreactivity at the kinase level, which is usually seen with nonselective Chk1/2 inhibitors such as AZD7762 [46], is rather unlikely to account for this effect, as SAR inhibits Chk1 with high specificity at the concentrations used here (Chk1 K_i_ = 13.3 nM, Chk2 K_i_ > 10 μM) [36]. Nevertheless, Chk1-dependent DNA repair of replication-induced DSB during S phase and of drug-induced DSB in the S and G2 phase takes place also in *p53*-wt cells, which together with some functional overlaps [47] most likely explains the inhibitory effects of SAR on both, *p53*-wt and -mut cells.

The efficiency of such treatments also in *p53*-wt glioblastoma patients is of high clinical relevance as, although about 30% of patients with primary and about 60% of patients with secondary glioblastoma have mutant *p53* [48, 49], intratumoural heterogeneity of *p53* mutation status has been reported and is thought to trigger tumour recurrence after p53-dependent treatment [50, 51]. However, it has to be kept in mind that enhanced adverse effects of Chk1 inhibitors on *p53*-wt normal tissue cells may occur if systemic DNA-damaging therapeutics are used.

It is therefore encouraging that no toxic effects of SAR-including single or multimodal treatment on neural progenitors occurred in our murine hippocampal slice model (Fig. 7d, e).

The specificity of the anti-clonogenic effects shown above was verified by concentration dependencies for 5-aza-dC, TMZ, SAR, and CCT244747, with and without single dose irradiation, at concentrations known to be reached or even exceeded in vivo (5-aza-dC: [52]; TMZ: [53]; SAR: [21]; CCT: [54]). Interestingly, radiosensitization by SAR was similar to that of the closely related [36] and orally available Chk1 inhibitor CCT244747 (Fig. 5a-d). Our findings go along with recent reports of radiosensitization by these specific Chk1 inhibitors in human lung, colon, and head and neck cancer cell lines and xenograft models (SAR: [18, 21]; CCT244747: [55]). Human primary glioblastoma cells showed a similar dose-dependent radiosensitization by SAR confirming the relevance of the results found in glioblastoma cell lines (Fig. 5**e**).

The lack of nestin/GFAP expression changes in human primary glioblastoma cells implicates no induction of cell differentiation by the treatments (Fig. 7a-c), although such responses have been described for 5-aza-dC in hypermethylated *IDH1*-mutant secondary gliomas [56].

In the future, the here documented effects of the ChK1 inhibitor SAR might be further enhanced by addition of other mediators. For example, ATR inhibitors are already entering clinical trials and could inhibit ATR-mediated phosphorylation and activation of Chk1, thereby lowering the threshold for induction of cell death by Chk1 inhibitors [57].

## Conclusion

Our data show that the inhibition of the S and the G2/M checkpoint by SAR may synergize with therapeutic settings involving different DNA-damaging sources such as irradiation, TMZ, or decitabine. Thereby, the Chk1-specific inhibition by SAR provides an effective opportunity to target especially p53-defective tumour cells and exerts a low risk for neurotoxicities.

## Data Availability

The datasets used and/or analyzed during the current study are available from the corresponding author on reasonable request.

## Abbreviations

5-aza-dC: 5-aza-2′-deoxycytidine, decitabine
ATR: Ataxia teleangiectasia and Rad3-related protein
Chk1: Checkpoint kinase 1
DNMT1: de novo methyltransferase 1
DSB: DNA double-strand breaks
EMA: European Medicines Agency
FDA: U. S. Food and Drug Administration
HR: Homologous recombination
IR: Irradiation, radiation therapy
NHEJ: Non-homologous end joining
OSF: Overall clonogenic survival
*p53*-mut: *p53*-mutated
*p53*-wt: *p53*-wildtype
pTPC: Potential tumor progenitor cells
SAR: SAR-020106
SF: Surviving fraction
TMZ: Temozolomide

## References

[1] Weller M, van den Bent M, Hopkins K, Tonn JC, Stupp R, Falini A (2014). EANO guideline for the diagnosis and treatment of anaplastic gliomas and glioblastoma. Lancet Oncol.

[2] Marcon F, Boei JJ, Natarajan AT (2000). Recombination between homologous chromosomes does not play a dominant role in the formation of radiation-induced chromosomal aberrations. Int J Radiat Biol..

[3] Lin Y, Lukacsovich T, Waldman AS (1999). Multiple pathways for repair of DNA double-strand breaks in mammalian chromosomes. Mol Cell Biol.

[4] Kastan MB, Kuerbitz SJ (1993). Control of G1 arrest after DNA damage. Environ Health Perspect.

[5] Perry ME, Levine AJ (1993). Tumor-suppressor p53 and the cell cycle. Curr Opin Genet Dev.

[6] Ohgaki H (2005). Genetic pathways to glioblastomas. Neuropathology..

[7] Zhang Ying, Dube Collin, Gibert Myron, Cruickshanks Nichola, Wang Baomin, Coughlan Maeve, Yang Yanzhi, Setiady Initha, Deveau Ciana, Saoud Karim, Grello Cassandra, Oxford Madison, Yuan Fang, Abounader Roger (2018). The p53 Pathway in Glioblastoma. Cancers.

[8] Wang X, Chen JX, Liu JP, You C, Liu YH, Mao Q (2014). Gain of function of mutant TP53 in glioblastoma: prognosis and response to temozolomide. Ann Surg Oncol..

[9] Moureau Sylvie, Luessing Janna, Harte Emma Christina, Voisin Muriel, Lowndes Noel Francis (2016). A role for the p53 tumour suppressor in regulating the balance between homologous recombination and non-homologous end joining. Open Biology.

[10] Gil Del Alcazar CR, Todorova PK, Habib AA, Mukherjee B, Burma S (2016). Augmented HR repair mediates acquired Temozolomide resistance in Glioblastoma. Mol Cancer Res.

[11] Sorensen CS, Hansen LT, Dziegielewski J, Syljuasen RG, Lundin C, Bartek J, Helleday T (2005). The cell-cycle checkpoint kinase Chk1 is required for mammalian homologous recombination repair. Nat Cell Biol.

[12] Liu Q, Guntuku S, Cui XS, Matsuoka S, Cortez D, Tamai K (2000). Chk1 is an essential kinase that is regulated by Atr and required for the G (2)/M DNA damage checkpoint. Genes Dev.

[13] Dillon MT, Good JS, Harrington KJ (2014). Selective targeting of the G2/M cell cycle checkpoint to improve the therapeutic index of radiotherapy. Clin Oncol.

[14] Dai Y, Grant S (2010). New insights into checkpoint kinase 1 in the DNA damage response signaling network. Clin Cancer Res.

[15] Gonzalez Besteiro MA, Gottifredi V (2015). The fork and the kinase: a DNA replication tale from a CHK1 perspective. Mutat Res Rev Mutat Res.

[16] Chen Z, Xiao Z, Gu WZ, Xue J, Bui MH, Kovar P (2006). Selective Chk1 inhibitors differentially sensitize p53-deficient cancer cells to cancer therapeutics. Int J Cancer.

[17] Qiu Z, Oleinick NL, Zhang J (2018). ATR/CHK1 inhibitors and cancer therapy. Radiother Oncol.

[18] Walton MI, Eve PD, Hayes A, Valenti M, de Haven BA, Box G (2010). The preclinical pharmacology and therapeutic activity of the novel CHK1 inhibitor SAR-020106. Mol Cancer Ther.

[19] Reader JC, Matthews TP, Klair S, Cheung KM, Scanlon J, Proisy N (2011). Structure-guided evolution of potent and selective CHK1 inhibitors through scaffold morphing. J Med Chem..

[20] Touchefeu Y, Khan AA, Borst G, Zaidi SH, McLaughlin M, Roulstone V (2013). Optimising measles virus-guided radiovirotherapy with external beam radiotherapy and specific checkpoint kinase 1 inhibition. Radiother Oncol..

[21] Borst GR, McLaughlin M, Kyula JN, Neijenhuis S, Khan A, Good J (2013). Targeted radiosensitization by the Chk1 inhibitor SAR-020106. Int J Radiat Oncol Biol Phys..

[22] Hagemann S, Heil O, Lyko F, Brueckner B (2011). Azacytidine and decitabine induce gene-specific and non-random DNA demethylation in human cancer cell lines. PLoS One.

[23] Patties I, Jahns J, Hildebrandt G, Kortmann RD, Glasow A (2009). Additive effects of 5-aza-2′-deoxycytidine and irradiation on clonogenic survival of human medulloblastoma cell lines. Strahlenther Onkol..

[24] Hoglund A, Nilsson LM, Forshell LP, Maclean KH, Nilsson JA (2009). Myc sensitizes p53-deficient cancer cells to the DNA-damaging effects of the DNA methyltransferase inhibitor decitabine. Blood..

[25] Maes K, De SE, Lemaire M, De RH, Menu E, Van VE (2014). The role of DNA damage and repair in decitabine-mediated apoptosis in multiple myeloma. Oncotarget.

[26] Deng T, Zhang Y (2009). Possible involvement of activation of P53/P21 and demethylation of RUNX 3 in the cytotoxicity against Lovo cells induced by 5-Aza-2′-deoxycytidine. Life Sci.

[27] Noushmehr H, Weisenberger DJ, Diefes K, Phillips HS, Pujara K, Berman BP (2010). Identification of a CpG island methylator phenotype that defines a distinct subgroup of glioma. Cancer Cell.

[28] Shinawi T, Hill VK, Krex D, Schackert G, Gentle D, Morris MR (2013). DNA methylation profiles of long- and short-term glioblastoma survivors. Epigenetics..

[29] Oppermann H, Dietterle J, Purcz K, Morawski M, Eisenloffel C, Muller W (2018). Carnosine selectively inhibits migration of IDH-wildtype glioblastoma cells in a co-culture model with fibroblasts. Cancer Cell Int.

[30] Quillien V, Lavenu A, Ducray F, Joly MO, Chinot O, Fina F, et al. Validation of the high-performance of pyrosequencing for clinical MGMT testing on a cohort of glioblastoma patients from a prospective dedicated multicentric trial. Oncotarget. 2016;7:61916–29. doi:10.18632/oncotarget.11322. 10.18632/oncotarget.11322 PMC5308700 27542245

[31] Encinas JM, Enikolopov G (2008). Identifying and quantitating neural stem and progenitor cells in the adult brain. Methods Cell Biol.

[32] Prager I, Patties I, Himmelbach K, Kendzia E, Merz F, Muller K (2016). Dose-dependent short- and long-term effects of ionizing irradiation on neural stem cells in murine hippocampal tissue cultures: Neuroprotective potential of resveratrol. Brain Behav.

[33] Gahwiler BH, Capogna M, Debanne D, McKinney RA, Thompson SM (1997). Organotypic slice cultures: a technique has come of age. Trends Neurosci.

[34] Patties I, Kortmann RD, Menzel F, Glasow A (2016). Enhanced inhibition of clonogenic survival of human medulloblastoma cells by multimodal treatment with ionizing irradiation, epigenetic modifiers, and differentiation-inducing drugs. J Exp Clin Cancer Res.

[35] Oppermann Henry, Purcz Katharina, Birkemeyer Claudia, Baran-Schmidt Rainer, Meixensberger Jürgen, Gaunitz Frank (2019). Carnosine’s inhibitory effect on glioblastoma cell growth is independent of its cleavage. Amino Acids.

[36] Matthews TP, Jones AM, Collins I (2013). Structure-based design, discovery and development of checkpoint kinase inhibitors as potential anticancer therapies. Expert Opin Drug Discov.

[37] Zhou L, Steller H (2003). Distinct pathways mediate UV-induced apoptosis in Drosophila embryos. Dev Cell..

[38] Urist M, Tanaka T, Poyurovsky MV, Prives C (2004). p73 induction after DNA damage is regulated by checkpoint kinases Chk1 and Chk2. Genes Dev.

[39] Matt S, Hofmann TG (2016). The DNA damage-induced cell death response: a roadmap to kill cancer cells. Cell Mol Life Sci.

[40] Syljuasen RG, Sorensen CS, Hansen LT, Fugger K, Lundin C, Johansson F (2005). Inhibition of human Chk1 increased initiation of DNA replication, phosphorylation of ATR targets, and DNA breakage. Mol Cell Biol.

[41] Liaw H, Lee D, Myung K (2011). DNA-PK-dependent RPA2 hyperphosphorylation facilitates DNA repair and suppresses sister chromatid exchange. PLos One.

[42] Bao S, Wu Q, McLendon RE, Hao Y, Shi Q, Hjelmeland AB (2006). Glioma stem cells promote radioresistance by preferential activation of the DNA damage response. Nature..

[43] Kim HJ, Kim JH, Chie EK, Young PD, Kim IA, Kim IH (2012). DNMT (DNA methyltransferase) inhibitors radiosensitize human cancer cells by suppressing DNA repair activity. Radiat Oncol..

[44] Moen EL, Stark AL, Zhang W, Dolan ME, Godley LA (2014). The role of gene body cytosine modifications in MGMT expression and sensitivity to temozolomide. Mol Cancer Ther.

[45] Hientz K, Mohr A, Bhakta-Guha D, Efferth T (2017). The role of p53 in cancer drug resistance and targeted chemotherapy. Oncotarget.

[46] Morgan MA, Parsels LA, Zhao L, Parsels JD, Davis MA, Hassan MC (2010). Mechanism of radiosensitization by the Chk1/2 inhibitor AZD7762 involves abrogation of the G2 checkpoint and inhibition of homologous recombinational DNA repair. Cancer Res.

[47] Yan S, Sorrell M, Berman Z (2014). Functional interplay between ATM/ATR-mediated DNA damage response and DNA repair pathways in oxidative stress. Cell Mol Life Sci.

[48] Kim H, Zheng S, Amini SS, Virk SM, Mikkelsen T, Brat DJ (2015). Whole-genome and multisector exome sequencing of primary and post-treatment glioblastoma reveals patterns of tumor evolution. Genome Res.

[49] Ohgaki H, Kleihues P (2007). Genetic pathways to primary and secondary glioblastoma. Am. J. Pathol.

[50] Jakovlevs A, Vanags A, Balodis D, Gardovskis J, Strumfa I (2014). Heterogeneity of Ki-67 and p53 expression in Glioblastoma. Acta Chirurgica Latviensis.

[51] Ren ZP, Olofsson T, Qu M, Hesselager G, Soussi T, Kalimo H (2007). Molecular genetic analysis of p53 intratumoral heterogeneity in human astrocytic brain tumors. J Neuropathol Exp Neurol..

[52] Aparicio A, Eads CA, Leong LA, Laird PW, Newman EM, Synold TW (2003). Phase I trial of continuous infusion 5-aza-2′-deoxycytidine. Cancer Chemother Pharmacol.

[53] Jackson S, Weingart J, Nduom EK, Harfi TT, George RT, McAreavey D, et al. The effect of an adenosine A2A agonist on intra-tumoral concentrations of temozolomide in patients with recurrent glioblastoma. Fluids Barriers CNS. 2018;15:2. doi:10.1186/s12987-017-0088-8. 10.1186/s12987-017-0088-8 PMC5767971 29332604

[54] Walton MI, Eve PD, Hayes A, Valenti MR, de Haven Brandon AK, Box G (2012). CCT244747 is a novel potent and selective CHK1 inhibitor with oral efficacy alone and in combination with genotoxic anticancer drugs. Clin Cancer Res.

[55] Patel R, Barker HE, Kyula J, McLaughlin M, Dillon MT, Schick U (2017). An orally bioavailable Chk1 inhibitor, CCT244747, sensitizes bladder and head and neck cancer cell lines to radiation. Radiother Oncol..

[56] Turcan S, Fabius AW, Borodovsky A, Pedraza A, Brennan C, Huse J (2013). Efficient induction of differentiation and growth inhibition in IDH1 mutant glioma cells by the DNMT Inhibitor Decitabine. Oncotarget.

[57] Massey AJ (2016). Inhibition of ATR-dependent feedback activation of Chk1 sensitises cancer cells to Chk1 inhibitor monotherapy. Cancer Lett.

